# Analytical Quality by Design-Compliant Development of a Cyclodextrin-Modified Micellar ElectroKinetic Chromatography Method for the Determination of Trimecaine and Its Impurities

**DOI:** 10.3390/molecules28124747

**Published:** 2023-06-13

**Authors:** Luca Marzullo, Roberto Gotti, Serena Orlandini, Patricie Slavíčková, Jakub Jireš, Michal Zapadlo, Michal Douša, Pavla Nekvapilová, Pavel Řezanka, Sandra Furlanetto

**Affiliations:** 1 Department of Chemistry “U. Schiff”, University of Florence, 50019 Sesto Fiorentino, Italy; luca.marzullo@unifi.it (L.M.); sandra.furlanetto@unifi.it (S.F.); 2 Department of Pharmacy and Biotechnology, University of Bologna, 40126 Bologna, Italy; roberto.gotti@unibo.it; 3 Zentiva, k.s., 10237 Prague, Czech Republic; patricie.slavickova@danone.com (P.S.); michal.zapadlo@zentiva.com (M.Z.); michal.dousa@zentiva.com (M.D.); 4 Department of Analytical Chemistry, Faculty of Chemical Engineering, UCT Prague, 16628 Prague, Czech Republic; pavla.nekvapilova@vscht.cz (P.N.); pavel.rezanka@vscht.cz (P.Ř.)

**Keywords:** capillary electrophoresis, experimental design, impurities, method operable design region, quality by design, trimecaine

## Abstract

In 2022, the International Council for Harmonisation released draft guidelines Q2(R2) and Q14, intending to specify the development and validation activities that should be carried out during the lifespan of an analytical technique addressed to assess the quality of medicinal products. In the present study, these recommendations were implemented in Capillary Electrophoresis method development for the quality control of a drug product containing trimecaine, by applying Analytical Quality by Design. According to the Analytical Target Profile, the procedure should be able to simultaneously quantify trimecaine and its four impurities, with specified analytical performances. The selected operative mode was Micellar ElectroKinetic Chromatography employing sodium dodecyl sulfate micelles supplemented with dimethyl-β-cyclodextrin, in a phosphate-borate buffer. The Knowledge Space was investigated through a screening matrix encompassing the composition of the background electrolyte and the instrumental settings. The Critical Method Attributes were identified as analysis time, efficiency, and critical resolution values. Response Surface Methodology and Monte Carlo Simulations allowed the definition of the Method Operable Design Region: 21–26 mM phosphate-borate buffer pH 9.50–9.77; 65.0 mM sodium dodecyl sulfate; 0.25–1.29% *v*/*v n*-butanol; 21–26 mM dimethyl-β-cyclodextrin; temperature, 22 °C; voltage, 23–29 kV. The method was validated and applied to ampoules drug products.

## 1. Introduction

Impurity profiling constitutes a fundamental part of Quality Control (QC) in pharmaceutical industries and consists of the detection and determination of impurities that may be present either in a drug substance (DS) or a drug product (DP). Impurities in a DP are represented by any compound other than the Active Pharmaceutical Ingredient (API) and its excipients [1]. Alongside Chromatography, Capillary Electrophoresis (CE) has been gaining a great reputation in this context due to its well-known advantages, relying on its high efficiency, wide versatility brought on by the several available operative modes, and the possibility of addition of many modifiers to the background electrolyte (BGE). Moreover, it presents the potential for simple implementation of enantioselective separations, which is of utmost importance in the case of chiral impurities analysis. Other outstanding advantages of CE are the minimal consumption of reagents and the limited use of organic solvents in favor of aqueous buffers, with consequently low environmental impact and low costs [1,2,3]. Different CE approaches have led to successful results in the field of impurity profiling, including Capillary Zone Electrophoresis, Micellar ElectroKinetic Chromatography (MEKC), MicroEmulsion Electrokinetic Chromatography, and Non-Aqueous Capillary Electrophoresis [1,4,5,6,7].

**Figure 1:**
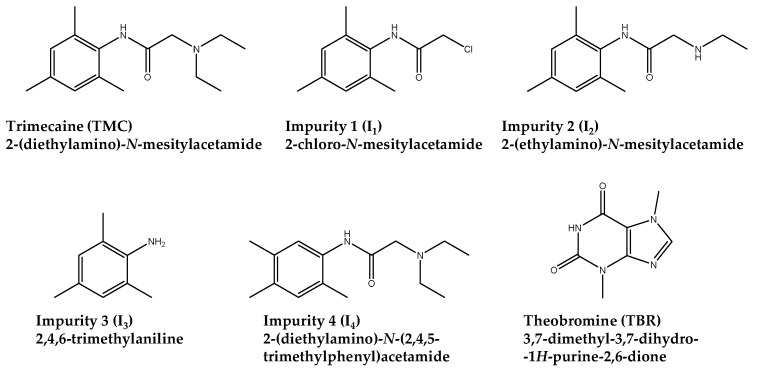
Molecular structures of the analytes and the internal standard theobromine.

Trimecaine hydrochloride (TMC) is an API used as a local anesthetic and cardiac antiarrhythmic of the acetanilide type. The Mesocain^®^ DP (Zentiva k.s. Prague, Czech Republic) is marketed through 10 mL injection solution ampoules, labeled to contain 100 mg TMC (10 mg mL^−1^). As reported by the drug product manufacturer, the main TMC impurities which can be found in the DP are mesityl chloroacetamide derivative (I_1_), desethyl trimecaine (I_2_), mesidine (I_3_) and 2,4,5-trimecaine (I_4_). The molecular structures of TMC and of the four impurities are reported in Figure 1.

A few methods can be found in the literature for the analysis of TMC in DP or biological samples. Determination in bulk or pharmaceutical preparations was performed by ion-sensitive electrodes [8,9,10], cyclic voltammetry [11], and HPLC with UV detection [12]. GC has been used for the simultaneous determination of local anesthetics, including TMC, in serum and plasma samples [13,14]. Regarding the CE technique, only Capillary Isotachophoresis was employed for the analysis of TMC and its demethylated metabolites in blood plasma [15]. To our knowledge, no analytical procedure has been reported yet for the simultaneous analysis of TMC and its impurities in DP. For TMC impurity profiling and assay, the manufacturer reports the use of a UPLC method with UV-Vis detection, whose details are reported in Supplementary Materials. 

Hence, the purpose of this study was to develop a CE method for the determination of TMC and its four main impurities to be employed for the routine QC of the DP. To carry out the development, an enhanced approach based on Analytical Quality by Design (AQbD) principles was applied, consisting of a comprehensive and rational strategy including the Design of Experiments (DoE) and Risk Assessment.

AQbD for Analytical Procedure (AP) development was first described a few years ago as a natural descendant of the wider concept of pharmaceutical Quality by Design (QbD). The QbD paradigm was introduced in the 2000s to assure the quality of pharmaceutical products and processes, with its cornerstones in the guidelines by the U.S. FDA [16] and International Council for Harmonisation (ICH) Q8 on Pharmaceutical Development [17]. Hence, the QbD concepts were applied to APs which are used for controlling the quality of the DP, giving rise to AQbD. From the first papers, forerunners in the field [18,19,20], through reviews/trends [21,22,23,24,25,26,27] to several specific pharmaceutical analysis applications, the interest in this topic has been rising steadily. In 2022 the turning point was the release of ICH draft guidelines Analytical Procedure Development Q14 [28] and Validation of Analytical Procedures Q2(R2) [29], to specify the development and validation activities that should be carried out during the lifespan of an AP used to assess the quality of medicinal products. In these documents, AQbD has finally received the official seal from regulatory bodies, and even if it is not yet compulsory for a new AP development, it will probably become so in the next future [26].

AQbD has been applied to pharmaceutical QC [23] and impurity profiling [30], and some applications in CE can be found in the literature for the control of both chiral and achiral API-related substances [31,32,33]. Due to the high number of Critical Method Parameters (CMPs) involved in CE optimization [34,35,36], AQbD is particularly useful as it allows an in-depth study of the effects of the CMPs on the analytical performances, particularly on the Critical Method Attributes (CMAs), up to the definition of the Method Operable Design Region (MODR). This is a multivariate zone where the requirements for the CMAs are achieved with a selected probability. In this study, the development of the CE method was carried out considering the new ICH draft guidelines and all the AQbD framework steps [21,35], up to method validation and application to real samples. 

## 2. Results and Discussion

The development of the CE method was structured, as previously extensively described in reviews and trends concerning the development of APs, by following AQbD principles [21,22] and considering the workflow recently highlighted by ICH guideline Q14 [28] adapted to CE methods [35]. The key steps consisted of the following phases: (i) definition of Analytical Target Profile (ATP); (ii) Knowledge Management; (iii) Risk Assessment; (iv) implementation of enhanced approach by Screening DoE; (v) implementation of enhanced approach by Response Surface Methodology (RSM); (vi) definition of MODR; (vii) Robustness and Method Control; (viii) Method Validation.

### 2.1. Analytical Target Profile

The ATP consists of the intended purpose of the AP. In this case, it was defined as the simultaneous quantitation of TMC and its impurities for the routine QC of injection solution in ampoules. The baseline resolution of the compounds in a low analysis time was required. The acceptance ranges for Validation Performance Criteria were as follows:

- for Selectivity, a complete resolution of the peaks of the analytes and no interference with the injection solution’s excipient;
- Quantitation Limit (QL) for the impurities ≤0.1% *w*/*w* with respect to the API;
- minimum Working Range for the impurities, from the QL to 1% with respect to the API test concentration, and minimum Working Range for the API, from 80 to 120% of the test concentration;
- with respect to Accuracy, measured recovery values included in the range of 98 to 102% for the API and in the range of 95 to 105% for the impurities;
- concerning Precision, evaluated in terms of Repeatability, RSD values within 2% for the API and within 5% for the impurities, with a higher accepted value at the QL (RSD ≤ 15%).

### 2.2. Knowledge Management

TMC, I_2_, I_3,_ and I_4_ are compounds with basic properties due to the presence of amino groups, whereas I_1_ is a neutral being the only nitrogen within an amide moiety (Figure 1). Thus, Knowledge Management was undertaken by running preliminary scouting experiments aimed at the selection of a proper separation system including an ionic pseudostationary phase.

The scouting phase consisted of the selection of a suitable combination of buffer and surfactant, as well as the evaluation of the possible addition of different modifiers, including a second surfactant, organic solvents, and cyclodextrins (CyDs). Additionally, the choice of the internal standard theobromine (TBR) was performed.

Initially, a simple pseudostationary phase made of 75 mM sodium dodecyl sulfate (SDS) micelles was tested as a starting separation system, setting standard values for voltage and temperature at 20 kV and 25 °C, respectively. Different types of buffer systems were evaluated, including 20 mM borate, 20 mM phosphate/borate, and 20 mM carbonate at pH 9.20, where the mixture of borate-phosphate led to the best results in terms of selectivity. 

Additionally, the possibility of using mixed micelles was examined, adding a second surfactant to SDS. Anyway, neither nonionic polyoxyethylene lauryl ether (Brij^®^ 35) nor zwitterionic 3-(*N*,*N*-dimethylmyristylammonio)propanesulfonate (MAPS) was useful to achieve an improvement of the electrophoretic pattern.

A favorable impact was obtained by adding a second pseudostationary phase, represented by either a neutral or an anionic CyD. Different CyDs, listed in Section 3.1, were added to the BGE at two different concentration values (10 and 25 mM), keeping the initial standard conditions as 20 mM phosphate/borate pH 9.20, 75 mM SDS. In all these experiments, the group of the first migrating peaks was represented by I_1_, I_2_, and I_3_, with the inversion of the migration of I_2_ and I_3_ in some cases. The last migrating peaks were TMC and finally I_4_. 

The best results were obtained by the addition of heptakis(2,6-di-*O*-methyl)-β-cyclodextrin (DMβCyD) and (2-hydroxypropyl)-γ-cyclodextrin (HPγCyD), obtaining a separation of all the peaks in about 10–12 min with a significant improvement in the selectivity with respect to the simple SDS system. Between the two CyDs, the separation of the two last migrating peaks (TMC and I_4_) using DMβCD was satisfying at both CyD concentration values, while for HPγCyD the complete separation was observed only at 25 mM, hence it was decided to choose the methylated one.

The separation system was completed by the addition of the organic modifier *n*-butanol, which was shown to lead to better peak shapes. The importance of *n*-butanol in perturbing equilibria in the presence of SDS micelles and thus effectively modulating the separation has been previously established [37].

### 2.3. Risk Assessment and Critical Method Parameters

After defining the fundamental separation system, Risk Assessment was carried out to establish the risk factors and thus define the CMPs, whose variation was expected to exert a significant influence on method performances. In particular, this phase was carried out by building a fishbone diagram, shown in Supplementary Figure S1, which made it possible to highlight the possible sources of variation, listed in four classes: Separation/Detection, Capillary, Injection, and BGE. Inside each class, the factors were further categorized into: (C) factors to be controlled; (N) noise parameters; (X) parameters that need experimental investigation to be fixed to a single value or to an acceptable range. From the scouting information, it was possible to select all the parameters related to Injection (5 s injection time at 50 mbar) and Capillary (uncoated, 48.5 cm total length, 40.0 cm effective length), as well as the type of buffer, surfactant, CyD, and organic modifier. On the other hand, CMPs, for which a more detailed study by DoE was needed, are highlighted in bold and were selected as the following: Voltage (V) and Temperature (T) within separation; concentration of phosphate/borate buffer (Buffer conc), buffer pH (pH), the concentration of SDS (SDS conc), the concentration of DMβCyD (CyD conc) and concentration of organic modifier (BuOH conc) within BGE.

### 2.4. Screening DoE

The effects of the CMPs selected through the fishbone diagram were first investigated in a Screening DoE, choosing their experimental domain based on the experiments run during Knowledge Management and previous experience, to maintain an acceptable generated current and a reasonable analysis time (Table 1).

Each factor was investigated at three levels so that a symmetric screening matrix 3^7^//16 was planned, as shown in Table 2. A Free-Wilson model [38] was hypothesized for relating the CMPs to the responses, represented by the CMAs. This model contains a constant term and two coefficients for each factor, namely the number of the levels studied (three) minus one:

*y* = *A*_0_ + *A*_1_*A* + *A*_2_*A* + *B*_1_*B* + *B*_2_*B* + *C*_1_*C* + *C*_2_*C* + *D*_1_*D* + *D*_2_*D* + *E*_1_*E* + *E*_2_*E* + *F*_1_*F* + *F*_2_*F* + *G*_1_*G* + *G*_2_*G* (1)

*y* represents the considered CMA, *A* is Buffer conc, *B* is pH, *C* is SDS conc, *D* is CyD conc, *E* is BuOH conc, *F* is V and *G* is T. The experiments were run, and the measured values of the responses are shown in Table 2.

Based on the ATP of the AP, the selection of the specific CMAs to be modeled was made by the direct observation of the obtained electropherograms, to identify all the critical points which could represent analytical issues. In particular, it was noticed that:

- the internal standard TBR and I_1_ were always the first and the second migrating peaks, respectively, and presented no separation issues;
- I_2_ was the third migrating peak in all the experiments, except for runs no. 1, nos. 3–6, and no. 10, where the inversion of the migration order occurred with respect to I_3_. Consequently, the resolution between I_2_ and I_3_ assumed both positive and negative values: a positive value when I_2_ was the first migrating peak (lower migration time) and I_3_ was the second migrating peak (higher migration time), a negative value when a change in migration order occurred, with I_3_ migrating before I_2_;
- Resolution between TMC and the first adjacent migrating peak (I_2_ or I_3_) was never critical and could be discarded from the data treatment;
- Efficiency of the I_2_ peak was critical with respect to the other peaks so it was included in data treatment;
- I_4_ was the last migrating peak in all the experiments and its migration time corresponded to analysis time.

In summary, the direction that the screening phase should be able to give to the subsequent optimization phase (RSM) was:

- To obtain a baseline resolution between all the peak pairs: R_1_ (TBR/I_1_); R_2_ (I_1_/I_2_); R_3_ (I_2_/I_3_); R_4_ (I_3_/TMC); R_5_ (TMC/I_4_). As mentioned above, for R_1_ and R_4_ no critical issues were observed and these responses were discarded from the data treatment;
- To constrain the R_3_ (I_2_/I_3_) resolution towards positive values, so that the final migration order is first I_2_ and then I_3_;
- To maximize I_2_ efficiency N_I2_ (I_2_ number of theoretical plates);
- To minimize analysis time t (calculated as I_4_ migration time).

To achieve these aims, the CMAs and their thresholds were fixed as follows: R_2_ ≥ 1.5; R_3_ ≥ 1.5; R_5_ ≥ 1.5; N_I2_ ≥ 10,000; t ≤ 10 min.

**Figure 2:**
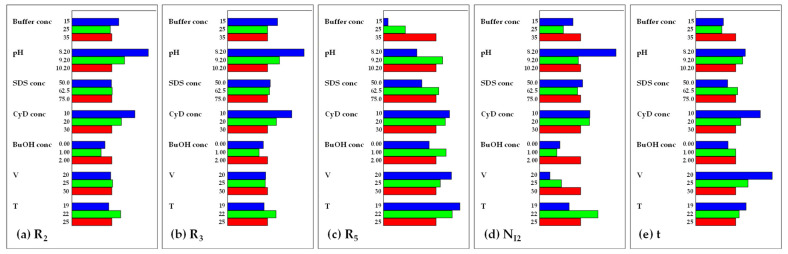
Screening graphic analysis of effects. (**a**) R_2_, resolution I_1_/I_2_; (**b**) R_3_, resolution I_2_/I_3_; (**c**) R_5_, resolution TMC/I_4_; (**d**) N_I2_, I_2_ efficiency; (**e**) t, analysis time.

After this preliminary evaluation of the responses and of the electropherograms, statistical treatment of the data was performed, and all five models for the CMAs were found significant by ANOVA. A graphic analysis of the effects was drawn, and the results are reported in Figure 2 and in Supplementary Figure S2. From Figure 2 it is possible to understand the effect of the levels of each CMP on the CMA. For every CMP three bars are shown, corresponding to the three levels tested; a longer bar corresponds to the maximization of the CMA (higher value), while a shorter bar corresponds to the minimization (lower value). From Supplementary Figure S2 it is possible to find out if changing the level from one to another there is a statistically significant effect on the CMA or not: the orange bars correspond to a significant difference. For instance, the bar known as b1/2–1 refers to the effect observed when moving from level 2 to level 1 of the first CMP, i.e., Buffer conc.

As for R_2_ and R_3_, the trend of the effects of the change in the considered factors was very similar in terms of both weight and sign. The most influencing factors were pH and CyD conc, for both of which the lower value resulted in the best one. As for Buffer conc, BuOH conc, and T, the best levels for each response were, respectively, the lower, the higher, and the medium one. Neither the change in SDS conc nor the change in V had a great influence on these CMAs; with the first being not significant, and the second having a negligible weight when compared to the other factors.

As for R_5_, the trend observed was quite different. The change in levels of all seven CMPs exerted a significant effect on this CMA. The increase of Buffer conc, pH, and SDS conc was found to have a favorable effect on this response, while the opposite was observed for CyD conc, V, and T, for which lower values led to its maximization. For BuOH conc, the medium level was preferred.

Additionally, concerning efficiency N_I2_ and analysis time, the graphs highlighted how each of the CMP had a significant effect, even if with different weights. In particular, the levels which led to a maximization of N_I2_ were a low value for pH, high values for BuOH conc and V, and a medium value for T; while the levels which led to a minimization of analysis time were a low value of Buffer conc and high values for both CyD conc and V.

Hence, considering the weight of the effects, SDS conc was fixed at 65.0 mM, namely next to the center of the experimental domain, in light of its relevant influence on the maximization of R_5_. The value of T was fixed at 22 °C, corresponding to the best value for increasing R_2_, R_3,_ and N_I2_, while also representing a good compromise between R_5_ and analysis time. As for the other factors, the experimental domain was moved towards a field that constituted an acceptable compromise among the responses, according to what is reported in Table 1, apart from V, for which the studied range remained the same.

### 2.5. Response Surface Methodology 

The RSM experimental domain selected for the CMPs is reported in Table 1. The following quadratic model was postulated linking the CMPs to the CMAs:

y = β_0_ + β_1_x_1_ + β_2_x_2_ + β_3_x_3_ + β_4_x_4_ + β_5_x_5_ + β_11_x_1_^2^ ++ β_22_x_2_^2^ + β_33_x_3_^2^ + β_44_x_4_^2^ + β_55_x_5_^2^ + β_12_x_1_x_2_ + β_13_x_1_x_3_ + β_14_x_1_x_4_ + β_15_x_1_x_5_ + β_23_x_2_x_3_ + β_24_x_2_x_4_ + β_25_x_2_x_5_ + β_34_x_3_x_4_ + β_35_x_3_x_5_ + β_45_x_4_x_5_ + ε (2)

where y is the CMA and the independent variables are listed as x_1_ (pH), x_2_ (Buffer conc), x_3_ (CyD conc), x_4_ (V), x_5_ (BuOH conc); β_0_ is the intercept, β_i_ are the linear coefficients, β_ii_ are the quadratic terms and β_ij_ the interaction coefficients; ε represents the experimental error. 

To estimate the coefficients of the model, an Orthogonal Central Composite Design was planned, where all the factors are studied at five levels (−α, −1, 0, +1, +α) with α = 1.66 [39]. The plan with the obtained responses is shown in Table 3.

It can be observed that, in this case, only in seven of the experiments did an inversion of the migration order between I_2_ and I_3_ occur (corresponding to negative values of R_3_). In three cases (no. 7, no. 13, no. 15) I_2_ and I_3_ peaks overlapped (R_3_ value was therefore reported equal to zero); hence, in these runs, it was not possible to measure N_I2_ efficiency (n.d. in Table 3).

Among the responses, only N_I2_ was transformed using a Logarithmic function (Log N_I2_), while all the others were treated as such. The models were refined by removing non-significant terms to obtain better values for goodness of prediction (Q^2^) and the quality parameters of the models were good for all the responses [40], ranging from 0.8257 to 0.9959 for R^2^ and from 0.4570 to 0.9823 for Q^2^ (Supplementary Table S1). Hence, it was possible to employ these models for optimization, drawing graphic analysis of effects and contour plots. 

A graphical analysis of effects is reported in Supplementary Figure S3. The examination of the graphs made it possible to obtain information at first glance on the weight and the sign of the coefficients of the CMA models; the longer the bar, the higher the weight of the effect, with the error bars representing the confidence interval. It could be highlighted that:

- pH was significant on all the CMAs and its most important linear effects were exerted on R_2_ and R_3_, although with opposite signs (negative for R_2_ and positive for R_3_, respectively);
- Buffer conc had a notable positive linear effect both on R_5_ and analysis time;
- CyD conc was significant in all the responses apart from N_I2_. The effect on resolution was the opposite: an increase in CyD conc leads to a minimization of R_2_ and a maximization of R_3_ and R_5_;
- Voltage has a predominantly negative effect on analysis time, as expected, but also a significant influence on R_2_;
- The effect of BuOH conc is significant, even if quite limited, on both R_3_, R_5,_ and analysis time;
- Quadratic effects were highlighted for pH, CyD conc, and V. The most important of them was the negative one, exerted by pH, on N_I2_;
- Significant interaction effects were found on R_3_, N_I2,_ and analysis time, but were all limited in their extent.

The contour plots are reported in Supplementary Figures S4–S8, showing the target and minimum/maximum values (CMA thresholds) considered acceptable. In these plots, CyD conc is plotted vs. pH at three different values of V (20, 25, and 30 kV) and Buffer conc (20, 25, 30 mM), while BuOH conc is fixed at 1.00% *v*/*v*. Principal results can be summarized thus:

- For R_2_, a wide zone of the domain led to acceptable predicted values, excluding the area corresponding to high pH values and high CyD conc (Supplementary Figure S4);
- Concerning R_3_, the plot was divided into distinct zones corresponding to negative or positive values of predicted response; the desired zone corresponded to high values of pH and high values of CyD conc, and the higher values of this CMA were obtained at low values of V (Supplementary Figure S5);
- Looking at R_5_, the target value was obtained throughout all the experimental domains. A curvature of the isoresponse lines was observed due to the presence of quadratic effects of both pH and CyD conc (Supplementary Figure S6);
- The same curvature was also observed for N_I2_, for which the target approximately corresponded to the zone located at the center of the domain of CyD conc and V (Supplementary Figure S7);
- For analysis time, as expected, the best results were located at high values of V, but the target could be achieved also at medium values (Supplementary Figure S8).

**Figure 3:**
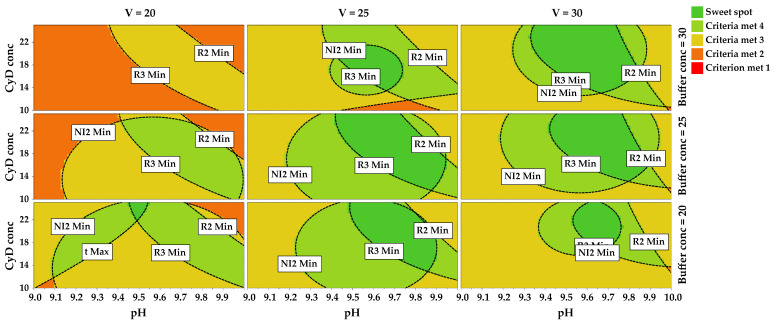
Sweet spot plot obtained reporting CyD conc vs. pH at three different values of V and Buffer conc, with BuOH conc fixed at 1.00% *v*/*v*.

Summarizing the information obtained by the contour plots and overlaying the predicted values for the five CMAs, the sweet spot plot shown in Figure 3 was obtained. According to the color scale reported in the legend, the sweet spot corresponds to the dark green zone, where all the CMA requirements are fulfilled; then, from light green to yellow, orange, and red color, it is possible to distinguish the zones where four, three, two or one CMA criteria are met, respectively. The wider sweet spot can be found in the plot corresponding to high values of V and high values of Buffer conc.

### 2.6. Method Operable Design Region

**Figure 4:**
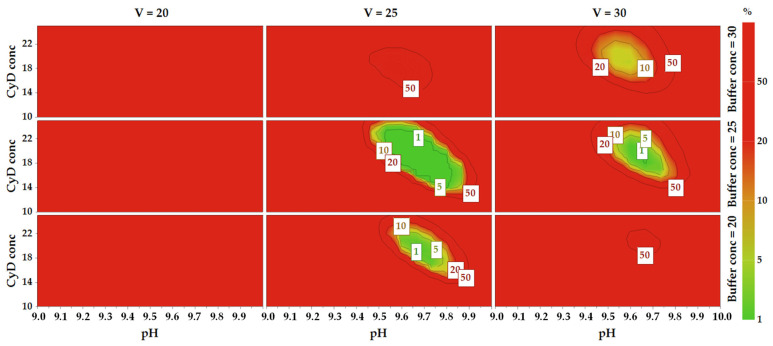
Probability maps obtained reporting CyD conc vs. pH at three different values of V and Buffer conc, with BuOH conc fixed at 1.00% *v*/*v*. The labels in the squares indicate the percentage risk of error. The MODR is colored green and is included in the 10% isoprobability lines.

The MODR was defined using Monte Carlo Simulations [41] combined with the calculated models, as the zone where the probability of fulfilling all the CMA requirements reaches a value ≥90% (risk ok error < 10%). The probability maps are reported in Figure 4, showing the risk of failure throughout the experimental domain, and the MODR corresponds to the green zone included in the 10% isoprobability line. If these maps are compared with the sweet spot plot, it is clear that when adding the model error in the predictions of the response distributions the situation drastically changes. The sweet spot quadrant matching high values of V and high values of Buffer conc no longer corresponds to the desired zone in the probability maps. The wider green zone in the probability maps is contained in the quadrant at medium values of V and Buffer conc.

**Figure 5:**
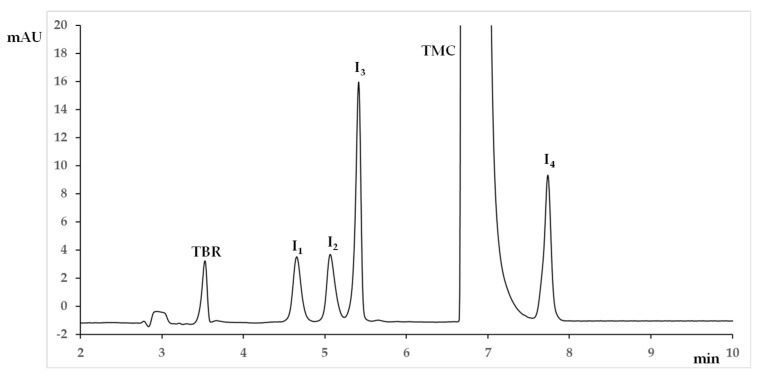
Electropherogram in the working point (See Table 1 for experimental conditions). TMC, Trimecaine; I_1_, Impurity 1; I_2_, Impurity 2; I_3_, Impurity 3; I_4_, Impurity 4; TBR, Theobromine.

The CMAs intervals corresponding to the MODR were the following: pH, 9.50–9.77; Buffer conc, 21–26 mM; CyD conc, 17–23 mM; V, 23–29 kV; BuOH conc, 0.25–1.29% *v*/*v* (Table 1). The MODR was validated by performing verification experiments at its edges, confirming that the CMA thresholds were not crossed. The working point for routine analysis was slightly adjusted from the original set point to fix simpler values to be set for the CMAs and corresponded to: Buffer conc, 23 mM; pH, 9.70; CyD conc, 20 mM; BuOH conc, 1.00%; V, 25 kV. When applying these conditions, the analysis time was lower than 8 min and the generated current was about 80 μA. The typical electropherogram is shown in Figure 5. 

### 2.7. Robustness and Method Control

According to ICH Q14, robustness should be assessed during method development [28]. Even if it was demonstrated that, inside the MODR, the CMA criteria are fulfilled with a certain probability, to achieve adequate proof that the procedure maintains suitable performances during normal use, all the CMPs considered in the screening phase were examined through a robustness study. A linear model was postulated for linking the CMPs to the CMAs, and a Plackett–Burman matrix was employed to estimate its coefficients. The experimental plan with the measured responses is shown in Supplementary Table S2. All the measured results fulfilled the criteria, even if SDS conc and T had not been included in the RSM for MODR definition.

A graphic analysis of the effects is shown in Supplementary Figure S9. No CMA had a significant effect on R_2_ and N_I2_, while pH and T were significant on R_3_, V, and T on R_5_ and V on t. Regarding V, a significant effect on analysis time is usually observed in an interval of 3 kV, which is the narrower range that could be selected for robustness testing due to technical reasons. The conclusion of this study indicated that particular attention should be paid to the proper control of pH when preparing the buffer solution for the BGE.

The Method Control for the AP was derived from a robustness study [21], defining System Suitability Test. When the AP is applied to a standard test mixture of the analytes in the working conditions, the CMA measured values should be included inside the acceptance intervals for the CMAs ranging from the lower values to the higher values observed when performing robustness testing: 1.92 < R_2_ < 3.20; 1.81 < R_3_ < 3.08; 3.14 < R_5_ < 4.77; 13,124 < N_I2_ < 24,082; 7.12 < t < 9.11. As for N_I2_, the interval was widened to a higher value to include the predicted values from the calculated N_I2_ model. Should the measured values fall outside these ranges, an investigation should be carried out to identify the reasons (instrumental, analytical operation, sample, BGE) which led to the out-of-specification values, corrective action should be taken and then a performance check on the AP should be carried out, to make sure that the procedure is back in specification.

### 2.8. Method Validation and Application 

Validation was performed according to ICH Q2(R1) [42], considering the indications furnished in draft guideline ICH Q2(R2) [29]. The measured Product Attributes were Purity (Quantitative) for the impurities and Assay Content for the API. The Performance Characteristics to be assessed were Selectivity, Working Range including Suitability of Calibration model and for impurities QL and Detection Limit (DL), Accuracy, and Precision in terms of Repeatability. The full validation data are reported in Supplementary Table S3.

Selectivity was demonstrated by the absence of interference with respect to the analyte peaks. The quantification of the compounds was not impacted by the presence of other components in the matrix and all the peaks were baseline separated (*n* = 4, α = 0.05): R_1_ (TBR/I_1_), 7.63 ± 0.24; R_2_ (I_1_/I_2_), 2.16 ± 0.06; R_3_ (I_2_/I_3_), 2.21 ± 0.07; R_4_ (I_3_/TMC), 5.33 ± 0.72; R_5_ (TMC/I_4_) 3.65 ± 0.28. The electropherogram obtained in the optimized conditions is reported in Figure 5.

As for the Working Range, a linear relationship between analyte concentration and the response was evaluated within the Working Range of the AP. The response was represented by the ratio between the corrected area (area/migration time) of the peak of the considered analyte and that of the internal standard. Six samples, each with two replicates, with concentration values appropriately distributed within the Working Range, were considered. Homoscedasticity was verified by the residual charts (not shown), observing that residuals showed no particular trend with the increase of the x-variable. Linearity data, including coefficient of determination R^2^, intercept, and slope of the linear regression, including their standard deviation and standard deviation of the residuals, are shown in Supplementary Table S3.

The Lower Range Limits for the impurities were estimated based on their signal-to-noise ratios, 3:1 for LD and 10:1 for QL. DL and QL ranged from 0.02% to 0.07% and from 0.06% to 0.10% of the API test concentration, respectively.

The Working Range for TMC was from 3 to 6 mg mL^−1^, corresponding to 60 to 120% of the test concentration. The Working Ranges for the impurities were from QL to 1% of the test concentration. Two replicates were made for each of the six concentration levels (*k* = 12), with TBR concentration constantly kept at 0.05 mg mL^−1^.

Accuracy was evaluated by applying the AP to an analyte of known purity and the measured vs. the theoretically expected result was evaluated. This parameter was assessed using four concentration values (QL-low-medium-high) with three replicates each, covering the Working Range, and was reported as the mean recovery percentage by the assay of a known added amount of analyte in the sample, with its confidence interval. The same samples were also used to evaluate Precision in terms of Repeatability. The confidence interval and RSD values of recoveries were then calculated. To obtain information on intermediate precision, as regards variations related to different days, instrumental repeatability for corrected peak areas and analysis time was assessed by performing six replicate injections of TMC and its impurities over 3 days. The results, reported as within-day RSD and total RSD, are shown in Supplementary Table S4. 

The method was applied to the analysis of two DS samples and two DP samples represented by Mesocain^®^ ampoules labeled to contain 100 mg 10 mL^−1^ TMC. Percentages of label claim were found in agreement both with the declared content and with the results obtained by the UPLC method used by the manufacturer (Supplementary Table S5). The electropherogram of the real sample of Mesocain^®^ is shown in Supplementary Figure S10. The presence of any impurity was not demonstrated at levels above the LOD. 

A comparison between the developed CE method and the UPLC method used by the manufacturer is shown in Supplementary Table S6, to highlight their main advantages and drawbacks. CE has better performances in terms of analysis time (8 min vs. 20 min) and the use of organic solvent is minimal (μL vs. mL). This leads not only to lower costs but also to lower environmental impact compared to UPLC. On the other hand, the performances of the UPLC method in terms of sensitivity are better, as this technique is capable of reaching both lower QL and DL values for all the impurities. 

## 3. Materials and Methods

### 3.1. Chemicals and Reagents

The reference standards of TMC and its related impurities I_1_, I_2_, I_3,_ and I_4_ were a gift from Zentiva (Prague, Czech Republic). TBR was purchased from Merck KGaA (Darmstadt, Germany) and was used as the internal standard. Mesocain^®^ injection solutions, labeled to contain 10 mg mL^−1^ TMC, were kindly supplied by Zentiva. A total of 86.1% phosphoric acid, boric acid, acetic acid, SDS, HPLC-grade methanol (MeOH), ethanol, *n*-butanol, Brij 35^®^, MAPS, and all other chemicals were obtained by Merck KGaA.

All the CyDs were acquired from Merck KGaA, apart from DMβCyD, which was purchased from Zentek srl (Milan, Italy). The tested CyDs included methyl-β-cyclodextrin (degree of substitution (D.S.) 1.5–2.1), heptakis(2,3,6-tri-*O*-methyl-β-CyD, (2-hydroxypropyl)-α-cyclodextrin (D.S. 0.6), (2-hydroxypropyl)-β-cyclodextrin (D.S. 0.6), HPγCyD (D.S. 0.6), (2-hydroxyethyl)-β-cyclodextrin (D.S. 0.7), carboxymethyl-β-cyclodextrin sodium salt, carboxyethyl-β-cyclodextrin sodium salt, sulfated-β-cyclodextrin sodium salt (D.S. 7–11).

Ultrapure water was used for preparing solutions and BGE was obtained through Millipore Simplicity 185 and Elix system (Billerica, MA, USA).

### 3.2. Solutions and Sample Preparation

10 mg mL^−1^ TMC stock solution and 1 mg mL^−1^ stock solutions of the impurities and the internal standard TBR were prepared in water/MeOH (1:1, *v*/*v*) and were kept at 4 °C for a week. Standard solutions were obtained by diluting the stock solutions with water in a vial of up to 500 μL.

Borate and phosphate/borate buffers were prepared by dilution of the proper amount of 0.5 M boric acid or 0.5 M boric acid/phosphoric acid mixture with water to about 80% of the final volume, then 1.0 M and 0.1 M NaOH solutions were used to reach the desired pH. Water was subsequently added to bring the solution up to the desired final volume. The pseudostationary phases (micelles and mixed micelles) were prepared by weighing the proper amount of surfactant or mixture of surfactants. An adequate amount of buffer was then added to the weighed powders. Organic modifiers and CyDs were subsequently added to the surfactant/s containing mixtures to obtain the desired BGEs.

For the preparation of the sample solution, the content of 5 ampoules of Mesocaine^®^ injection solution, labeled to contain 10 mg mL^−1^ TMC, was mixed; 250 μL of the mixture were introduced in a vial for the analysis, then 150 μL of methanol and 75 µL of ultrapure water were added, together with 25 μL of TBR standard solution. The final sample solution contained 5 mg mL^−1^ TMC and 0.05 mg mL^−1^ of the internal standard TBR.

### 3.3. CE Instrumentation and Analysis

CE analyses were run on an Agilent 7100 Capillary Electrophoresis System with a UV-Vis detector (Agilent Technologies, Waldbronn, Germany). A total of 50 μm fused silica capillaries (375 μm outer diameter) were purchased from CM Scientific Ryefield (Dublin, Ireland) and were cut to a total and effective length of 48.5 and 40.0 cm, respectively. UV detection was carried out at 210 nm and the separations were all carried out in positive polarity mode. The temperature was set at 22 °C. Hydrodynamic injection of the sample was performed in two steps, applying first 50 mbar pressure for 5 s to the sample injection vial, then 50 mbar for 5 s to a buffer-filled vial, for plug injection.

Each new capillary was rinsed with 1 M NaOH, 0.1 M NaOH, and water for 5 min each. Daily, the capillary was rinsed with 0.1 M NaOH and water for 2 min each before use. Between the runs, the conditioning was made as follows: MeOH (1 min), 0.1 M NaOH (1 min), water (1 min), and BGE (3 min).

The optimized CE conditions corresponded to the following, with the MODR in brackets: capillary total length, 48.5 cm (effective length, 40.0 cm); temperature, 22 °C; voltage, 25 kV (23–29 kV); BGE: 23 mM (21–26 mM) phosphate-borate buffer pH 9.70 (9.50–9.77), 65.0 mM SDS, 1.00% *v*/*v* (0.25–1.29% *v*/*v*) *n*-butanol, 20 mM (21–26 mM) DMβCD. 

### 3.4. Data Analysis and Software

OpenLab CDS ChemStation Edition (Agilent Technologies) was used for instrument control, electropherograms acquisition, and data analysis.

The screening phase was carried out by employing a symmetric screening matrix generated by NemrodW software (NemrodW, LPRAI sarl, Marseille, France), with which related data analysis and graphical analysis of effects were performed.

The optimization phase, including RSM and MODR identification using Monte Carlo Simulations [41], was carried out by MODDE 13 software (Sartorius Data Analytics AB, Göttingen, Germany), which allowed contour plots and probability maps to be drawn. The MODR was defined by setting the target for defect per million opportunities (DPMO) at 100,000, namely to a risk of error equal to 10% [40].

## 4. Conclusions

In this study, the development of a CE method for the simultaneous analysis of TMC and its impurities in DS and DP was carried out using an AQbD approach. The application of AQbD made it possible to identify the risk factors that could lead to AP failure and to achieve a thorough understanding of the effects of the CMPs on the method’s performance. Having defined the ATP, Knowledge Management was used to address the selection of the CE operative mode, which was chosen as CyD-MEKC with the addition of *n*-butanol as an organic modifier. A screening phase was carried out using a symmetric screening matrix; then RSM by Orthogonal Central Composite Design, combined with Monte Carlo Simulations, led to the definition of the MODR. Despite the MODR being identified as a robust zone for the AP with a selected probability, robustness testing highlighted the need to carefully control the pH when preparing the BGE. Though the road ahead for AQbD to be fully embraced by both academic and industrial researchers is still long, examples of procedure development inside an AQbD framework, such as the one herein presented, can offer the opportunity to consolidate and deepen the knowledge in this field, to be ready to comply with the most recent guidelines in the analytical pharmaceutical field: ICH Q14 and Q2(R2). 

## Figures and Tables

**Table 1:** Critical Method Parameters for the CE method.

CMPs	CMP Abbreviation	Screening Levels	RSM Levels and Domain ^1^	Working Point with MODR
Buffer concentration	Buffer conc	15–25–35 mM	20–30 mM	23 mM (21–26 mM)
Buffer pH	pH	8.20–9.20–10.20	9.00–10.00	9.70 (9.50–9.77)
SDS concentration	SDS conc	50.0–62.5–75.0 mM	65.0 mM	65.0 mM
DMβCyD concentration	CyD conc	10–20–30 mM	10–25 mM	20 mM (17–23 mM)
*n*-butanol concentration	BuOH conc	0.00–1.00–2.00% *v*/*v*	0.20–1.80% *v*/*v*	1.00% *v*/*v* (0.25–1.29% *v*/*v*)
Voltage	V	20–25–30 kV	20–30 kV	25 kV (23–29 kV)
Temperature	T	19–22–25 °C	22 °C	22 °C

^1^ RSM levels: CMPs levels which were kept constant in RSM; RSM domain: CMPs experimental range which was investigated in RSM.

**Table 2:** 3^7^//16 Symmetric screening matrix used in the screening phase.

No. Exp.	Buffer Conc (mM)	pH	SDS Conc (mM)	CyD Conc (mM)	BuOH Conc (% *v*/*v*)	V (kV)	T (°C)	R_2_	R_3_	R_5_	N_I2_	*t* (min)
1	15	9.20	62.5	30	1.00	30	25	1.67	−0.88	2.64	13,279	4.38
2	35	8.20	62.5	20	2.00	25	25	14.38	11.06	3.78	36,880	10.65
3	35	10.20	50.0	20	1.00	30	22	1.00	−2.25	4.87	29,692	5.22
4	25	10.20	75.0	10	1.00	25	25	2.53	−1.67	3.78	7550	9.51
5	35	9.20	75.0	30	0.00	25	22	3.83	−1.48	4.82	18,090	8.04
6	25	10.20	62.5	30	2.00	20	22	0.87	−3.64	4.09	14,510	10.56
7	25	9.20	75.0	20	2.00	30	19	4.58	0.00	4.32	19,078	6.81
8	25	9.20	62.5	20	1.00	25	22	5.65	1.38	4.78	12,229	8.35
9	35	9.20	62.5	10	1.00	20	19	5.81	2.74	7.14	3195	20.68
10	15	10.20	62.5	20	0.00	25	19	1.30	−2.50	3.23	6706	6.99
11	15	8.20	75.0	20	1.00	20	22	15.37	14.58	3.16	27,893	13.32
12	25	8.20	50.0	30	1.00	25	19	5.94	3.16	2.74	14,257	7.11
13	15	9.20	50.0	10	2.00	25	22	16.04	15.22	2.93	27,987	10.38
14	25	8.20	62.5	10	0.00	30	22	18.87	19.85	2.80	36,535	8.61
15	25	9.20	50.0	20	0.00	20	25	2.83	0.00	2.99	3809	8.91
16	25	9.20	62.5	20	1.00	25	22	5.34	1.10	4.64	12,342	8.10

R_2_, resolution I_1_/I_2_; R_3_, resolution I_2_/I_3_; R_5_, resolution TMC/I_4_; N_I2_, I_2_ efficiency; *t*, analysis time.

**Table 3:** Orthogonal Central Composite Design used in Response Surface Methodology.

No. Exp.	pH	Buffer Conc (mM)	CyD Conc (mM)	V (kV)	BuOH Conc (% *v*/*v*)	R_2_	R_3_	R_5_	N_I2_	*t* (min)
1	9.20	22	13.0	22	1.48	6.35	−3.43	2.47	8400	9.23
2	9.80	22	13.0	22	0.52	3.70	1.45	3.05	18,908	10.17
3	9.20	28	13.0	22	0.52	5.82	−2.60	3.37	7954	10.41
4	9.80	28	13.0	22	1.48	2.60	2.43	4.08	8604	11.89
5	9.20	22	22.0	22	0.52	4.47	−0.86	3.31	6884	8.68
6	9.80	22	22.0	22	1.48	1.81	3.32	4.74	12,511	9.54
7	9.20	28	22.0	22	1.48	5.38	0.00	5.20	n.d.^1^	9.79
8	9.80	28	22.0	22	0.52	1.63	3.11	4.79	8061	9.84
9	9.20	22	13.0	28	0.52	4.10	−1.77	2.94	4379	6.43
10	9.80	22	13.0	28	1.48	2.226	1.73	3.67	9325	6.86
11	9.20	28	13.0	28	1.48	4.09	−1.03	4.00	5713	7.35
12	9.80	28	13.0	28	0.52	2.54	1.37	3.06	8342	7.26
13	9.20	22	22.0	28	1.48	4.34	0.00	4.46	n.d.^1^	6.17
14	9.80	22	22.0	28	0.52	1.59	2.34	3.80	9707	5.80
15	9.20	28	22.0	28	0.52	4.48	0.00	3.41	n.d.^1^	6.32
16	9.80	28	22.0	28	1.48	1.42	3.13	4.92	13,364	6.66
17	9.00	25	17.5	25	1.00	4.40	−1.66	3.61	5076	7.79
18	10.00	25	17.5	25	1.00	1.51	2.73	4.53	6878	8.25
19	9.50	20	17.5	25	1.00	3.71	1.15	2.26	25,460	7.41
20	9.50	30	17.5	25	1.00	2.97	1.59	3.90	10,100	8.66
21	9.50	25	10.0	25	1.00	5.31	−0.95	4.31	17,255	9.29
22	9.50	25	25.0	25	1.00	2.26	2.29	3.96	12,609	7.41
23	9.50	25	17.5	20	1.00	4.51	1.33	3.20	26,097	11.39
24	9.50	25	17.5	30	1.00	2.92	1.27	3.77	19,239	5.62
25	9.50	25	17.5	25	0.20	4.02	0.89	2.36	26,779	7.47
26	9.50	25	17.5	25	1.80	2.87	1.63	3.91	10,783	8.37
27	9.50	25	17.5	25	1.00	3.35	1.32	3.06	17,716	7.91
28	9.50	25	17.5	25	1.00	3.56	1.36	2.98	18,518	8.27
29	9.50	25	17.5	25	1.00	3.85	1.27	3.06	23,917	8.04

^1^ n.d. = not determined.

## Data Availability

The data presented in this study are available from the corresponding author upon reasonable request.

## Supplementary Materials

The following supporting information can be downloaded at: https://www.mdpi.com/article/10.3390/molecules28124747/s1, Figure S1: Fishbone diagram; Figure S2: Screening graphic analysis of effects; Figure S3: RSM graphic analysis of effects; Figure S4: Resolution R_2_ contour plot; Figure S5: Resolution R_3_ contour plot; Figure S6: Resolution R_5_ contour plot; Figure S7: Efficiency N_I2_ contour plot; Figure S8: Analysis time contour plot; Figure S9: Robustness graphic analysis of effects; Figure S10: Electropherogram of the real sample Mesocain^®^; Table S1: Quality parameters of the RSM models; Table S2: Plackett–Burman Design for robustness testing; Table S3: Validation data; Table S4: Within-day and between-day instrumental repeatability; Table S5: Real sample analysis; Table S6: Comparison between the CE method and the UPLC method.

## Sample Availability

Not available.

## Abbreviations

AP—Analytical Procedure; API—Active Pharmaceutical Ingredient; AQbD—Analytical Quality by Design; ATP – Analytical Target Profile; BGE—Background Electrolyte; Brij 35^®^—Polyoxyethylene lauryl ether; CE—Capillary Electrophoresis; CMA—Critical Method Attribute; CMP—Critical Method Parameter; CyD—Cyclodextrin; DL—Detection Limit; DoE—Design of Experiments; DMβCyD—Heptakis(2,6-di-*O*-methyl)-β-cyclodextrin; DS—Drug Substance; DP—Drug Product; HPγCyD—Hydroxypropyl-γ-cyclodextrin; ICH—International Council for Harmonisation; MAPS—3-(*N*,*N*-dimethylmyristylammonio)propanesulfonate; MeOH—Methanol; MEKC—Micellar ElectroKinetic Chromatography; MODR—Method Operable Design Region; QbD—Quality by Design; QC—Quality Control; QL—Quantitation Limit; RSM—Response Surface Methodology; SDS—Sodium Dodecyl Sulfate; TBR—Theobromine; TMC—Trimecaine hydrochloride.
