# Supplementary material for: Analytical Quality by Design-Compliant Development of a Cyclodextrin-Modified Micellar ElectroKinetic Chromatography Method for the Determination of Trimecaine and Its Impurities
Source: Molecules. 2023 Jun 13;28(12):4747. doi: 10.3390/molecules28124747 (PMC10302722; doi:10.3390/molecules28124747)
Supplement: Supplementary file 1 [file molecules-28-04747-s001.zip › molecules-2431535-supplementary.pdf]

Supplementary Materials

Analytical Quality by Design-Compliant Development of a Cyclodextrin-Modified Micellar ElectroKinetic Chromatography Method for the Determination of Trimecaine and Its Impurities

Luca Marzullo [1], Roberto Gotti [2], Serena Orlandini [1,*], Patricie Slavíčková [3], Jakub Jireš [3,4], Michal Zapadlo [3], Michal Douša [3], Pavla Nekvapilová [4], Pavel Řezanka [4] and Sandra Furlanetto [1]

[1] Department of Chemistry "U. Schiff", University of Florence, 50019 Sesto Fiorentino, Italy; luca.marzullo@unifi.it (L.M.); sandra.furlanetto@unifi.it (S.F.)
[2] Department of Pharmacy and Biotechnology, University of Bologna, 40126 Bologna, Italy; roberto.gotti@unibo.it
[3] Zentiva, k.s., 10237 Prague, Czech Republic; patricie.slavickova@danone.com (P.S.); michal.zapadlo@zentiva.com (M.Z.); michal.dousa@zentiva.com (M.D.)
[4] Department of Analytical Chemistry, Faculty of Chemical Engineering, UCT Prague, 16628 Prague, Czech Republic; pavla.nekvapilova@vscht.cz (P.N.); pavel.rezanka@vscht.cz (P.Ř.)
* Correspondence: serena.orlandini@unifi.it

UPLC analysis with UV detection

1. Assay

Instrument: UPLC/UHPLC system with PDA detector

Chemicals: *Acetonitrile R1, Methanol R2, Water for chromatography R, Perchloric acid R*

Column: *Stationary phase*: ACQUITY UPLC CSH C18 (1.7 µm); length = 150 mm, internal diameter 3.0 mm; *Temperature*: 45 °C

Mobile phase: A: Dilute 0.6 ml of *Perchloric acid R* in 1000 ml of *Water for chromatography R.*
B: *Acetonitrile R1* / *Methanol R2* 600 / 400 (V/V)

Elution: linear gradient

The following elution gradient was used: time [min] 0–17.0–17.5–18.0–20.0; mobile phase B [% v/v] 10–70–70–10–10.

Flow rate: 0.70 ml/min

Detection: spectrophotometer at 263 nm

Recommendation for PDA detector: use 4.8 nm resolution of λ

Injection: 2 µl

Autosampler temperature: 15 °C

Data acquisition time: 17.5 min

Sample solvent: *Water for Chromatography R* / *Acetonitrile R* 50 / 50 (V/ V)

Sample solution: Accurately weigh about 50 mg of the examined substance and add 25 ml of *Acetonitrile R1* and 5 ml of *Water for Chromatography R*, insert into an ultrasonic bath for 5 min. After cooling to laboratory temperature dilute to 50.0 ml with *Water for Chromatography R*.

Reference solution: Accurately weigh about 50 mg of the *Trimecaine Hydrochloride – reference substance* and add 25 ml of *Acetonitrile R1* and 5 ml of *Water for Chromatography R*, insert into an ultrasonic bath for 5 min. After cooling to laboratory temperature dilute to 50.0 ml with *Water for Chromatography R*.

Typical retention time: *Trimecaine* about 5.5 min

System suitability:
System repeatability: relative standard deviation of the peak areas corresponding to *Trimecaine* in the chromatograms of five replicate injections of the reference solution is not more than 0.73 %.
Plate number: not less than 25 000 for the peak corresponding to *Trimecaine* in the chromatogram of the reference solution.

Evaluation:
Calculate the assay of *Trimecaine Hydrochloride* dried substance (X) in per cent according to the formula:

$$X(\%) = \frac{A_{test} \times m_{ref} \times P \times 100}{A_{ref} \times m_{test} \times (100 - s)}; \quad where$$

A_{test} is area of the peak corresponding to *Trimecaine* in the chromatogram of the sample solution
A_{ref} is area of the peak corresponding to *Trimecaine* in the chromatogram of the reference solution
m_{test} is weight of the examined substance in mg
m_{ref} is weight of *Trimecaine Hydrochloride – reference substance* in mg

P	is potency of *Trimecaine Hydrochloride – reference substance* in %
s	is loss on drying in the examined substance in %.

2. Related substances

The LC method is used for determination of related substances Impurity 1 (I_1), Impurity 2 (I_2), Impurity 3 (I_3), Impurity 4 (I_4), and unspecified impurities in *Trimecaine Hydrochloride*.

Instrument: UPLC/UHPLC system with UV/VIS or PDA detector

Chemicals: *Acetonitrile R1*, *Methanol R2*, *Water for chromatography R*, *Perchloric acid R*

Column: *Stationary phase*: ACQUITY UPLC CSH C18 (1.7 µm); length = 150 mm, internal diameter 3.0 mm; *Temperature*: 45 °C

Mobile phase: A: Dilute 0.6 ml of *Perchloric acid R* in 1000 ml of *Water for chromatography R*.
B: *Acetonitrile R1 / Methanol R2* 600 / 400 (V/V)

Elution: linear gradient
The following elution gradient was used: time [min] 0–17.0–17.5–18.0–20.0; mobile phase B [% v/v] 10–70–70–10–10.

Flow rate: 0.70 ml/min

Detection: spectrophotometer at 210 nm

Injection: 1 µl

Autosampler temperature:15 °C

Data acquisition time: 17.5 min

Sample solvent: *Water for Chromatography R / Acetonitrile R* 50 / 50 (V/V)

Sample solution: Accurately weigh about 50 mg of the examined substance and add 25 ml of *Acetonitrile R1* and 5 ml of *Water for Chromatography R*, insert into an ultrasonic bath for 5 min. After cooling to laboratory temperature dilute to 50.0 ml with *Water for Chromatography R*.

Reference solution: Accurately weigh about 50 mg of the *Trimecaine Hydrochloride – reference substance* and add 25 ml of *Acetonitrile R1* and 5 ml of *Water for Chromatography R*, insert into an ultrasonic bath for 5 min. After cooling to laboratory temperature dilute to 50.0 ml with *Water for Chromatography R*. Dilute 1.0 ml of this solution into 100.0 ml with the sample solvent. Dilute 0.8 ml of this solution into 10.0 ml with the sample solvent (concentration of the reference solution is 0.08% related to the sample solution).

Typical retention time: *Trimecaine* About 5.5 min

Relative retention time: (relative to *Trimecaine*)

Impurity 3 (I_3)	RRT 0.70
Impurity 2 (I_2)	RRT 0.85
Impurity 4 (I_4)	RRT 1.14
Impurity 1 (I_1)	RRT 1.87

System suitability:

System repeatability: relative standard deviation of the peak areas corresponding to Trimecaine in the chromatograms of five replicate injections of the reference solution is not more than 5.0%.

S/N ratio: signal to noise ratio of the peak corresponding to Trimecaine in the chromatogram of each injection of the reference solution is not less than 30.
Plate number: not less than 100 000 for the peak corresponding to Trimecaine in the chromatogram of the reference solution.

Evaluation:
Calculate the percentage content of individual impurities in dried substance (x_i) according to the formula:

$$x_i(\%) = \frac{A_{imp} \times m_{ref} \times C_f \times P \times 0.08}{A_{ref} \times m_{test} \times (100 - s)} \quad , where:$$

A_{imp} is area of the peak corresponding to individual impurity in the chromatogram of the sample solution
A_{ref} is area of the peak corresponding to Trimecaine in the chromatogram of the reference solution
m_{test} is weight of the examined substance in mg
m_{ref} is weight of *Trimecaine Hydrochloride* – reference substance in mg
C_f is correction factor of individual impurities (relative to *Trimecaine Hydrochloride*):
Impurity 3 (I_3), 0.98; Impurity 2 (I_2), 0.79; Impurity 4 (I_4), 0.76; Impurity I (I_1), 0.73.
P is potency of *Trimecaine Hydrochloride – reference substance* in %
s is loss on drying of the examined substance in %.

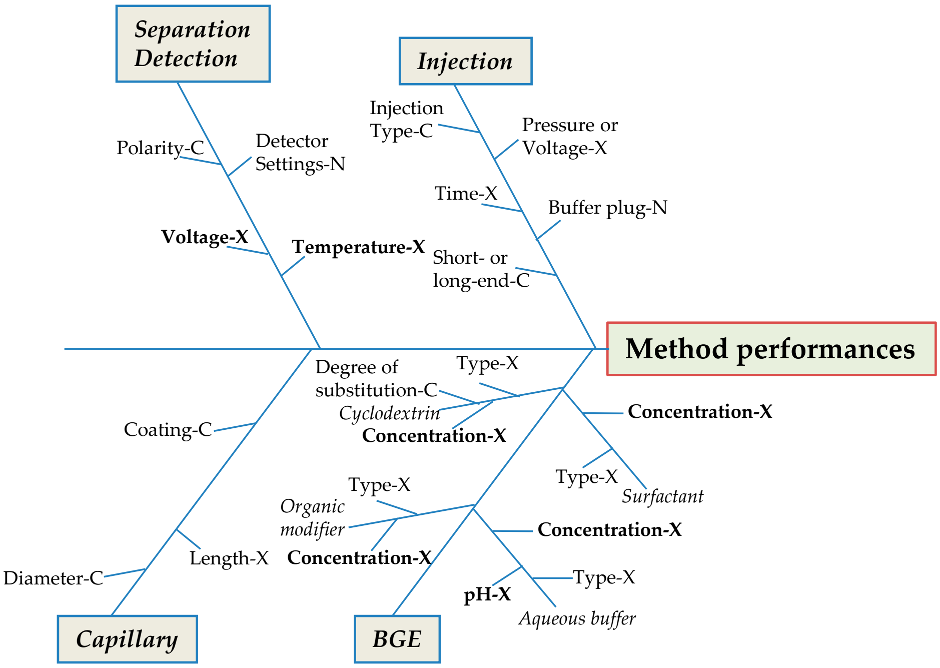

Figure S1. Fishbone diagram.

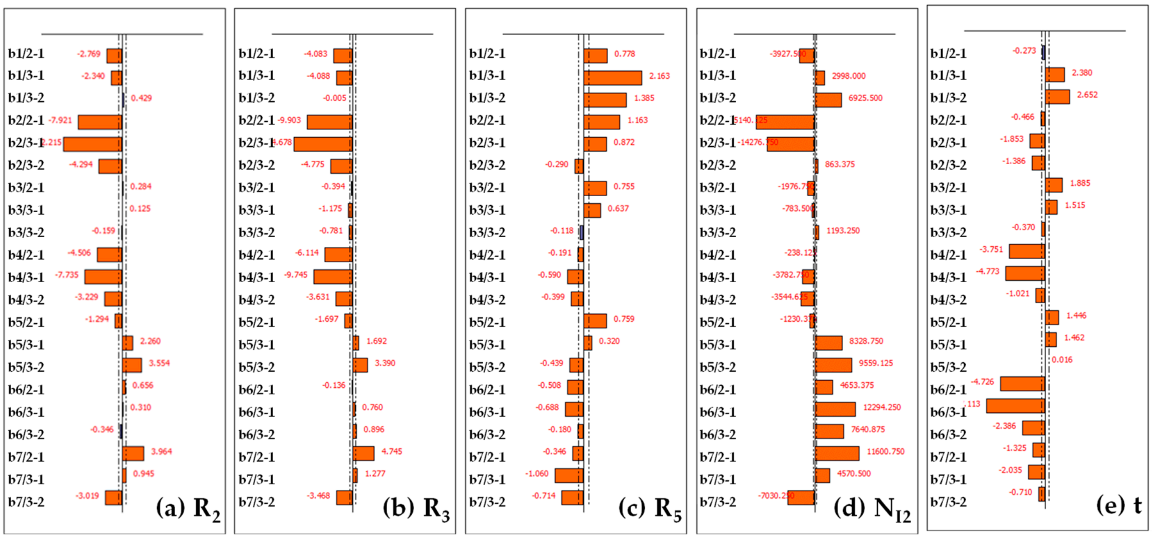

Figure S2. Screening graphic analysis of effects. (a) R_2, resolution I_1/I_2; (b), R_3, resolution I_2/I_3; R_5, resolution TMC/I_4; N_{I2}, I_2 efficiency; (e) t, analysis time. The orange bars indicate a change between two levels which has a significant effect on the corresponding CMA.

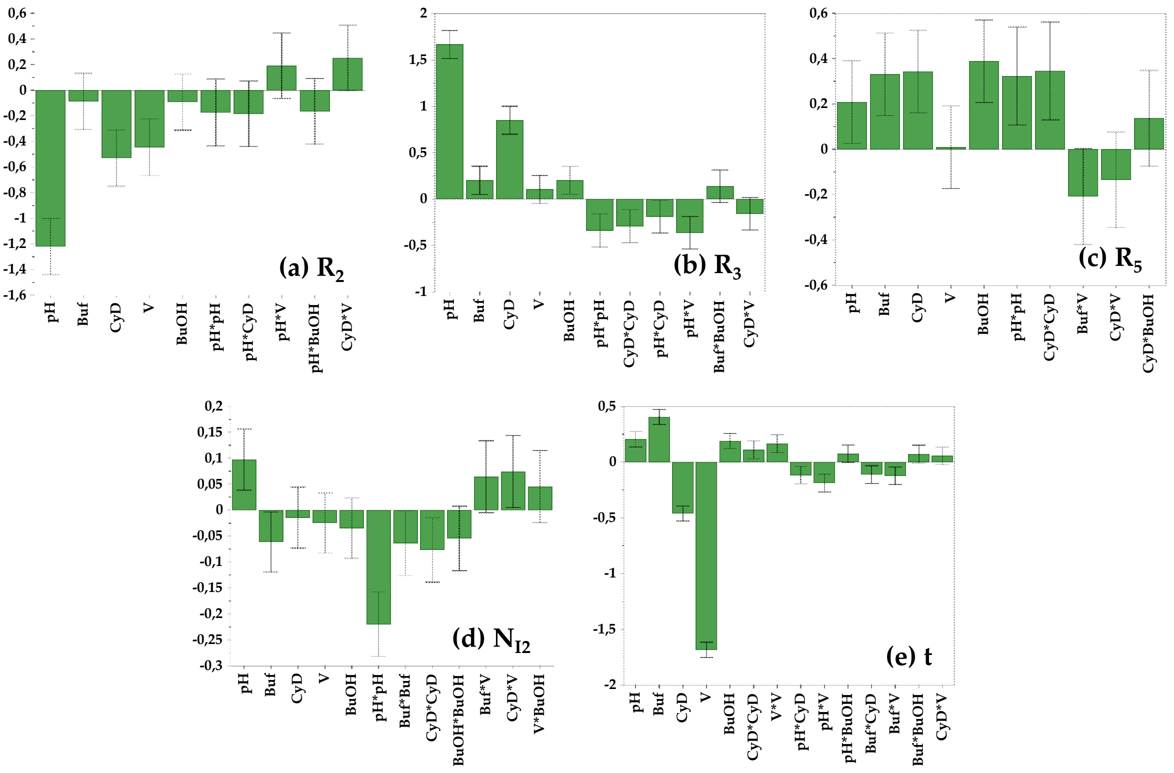

Figure S3. RSM graphic analysis of effects. (a) R_2, resolution I_1/I_2; (b), R_3, resolution I_2/I_3; (c) R_5, resolution TMC/I_4; (d) N_{I2}, I_2 efficiency; (e) t, analysis time.

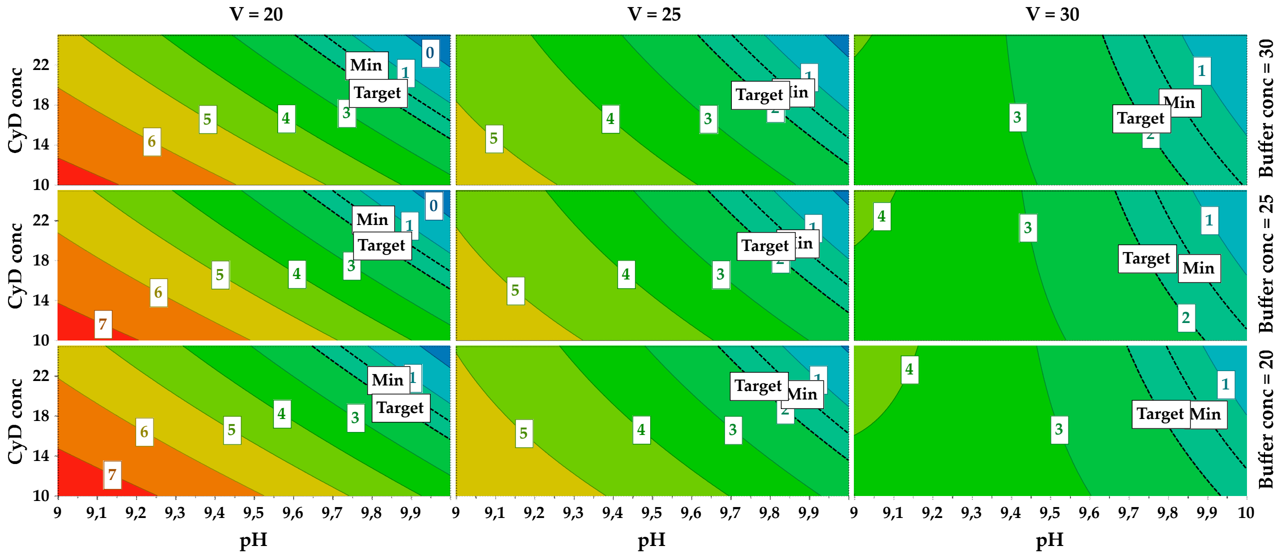

Figure S4. Resolution R_2 contour plot.

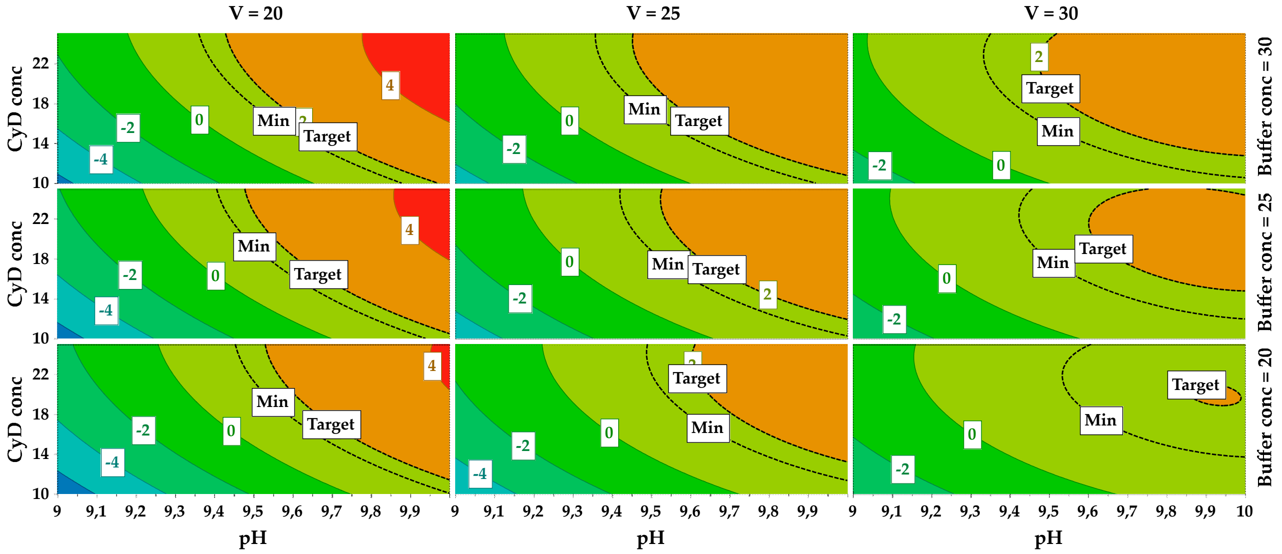

Figure S5. Resolution R3 contour plot.

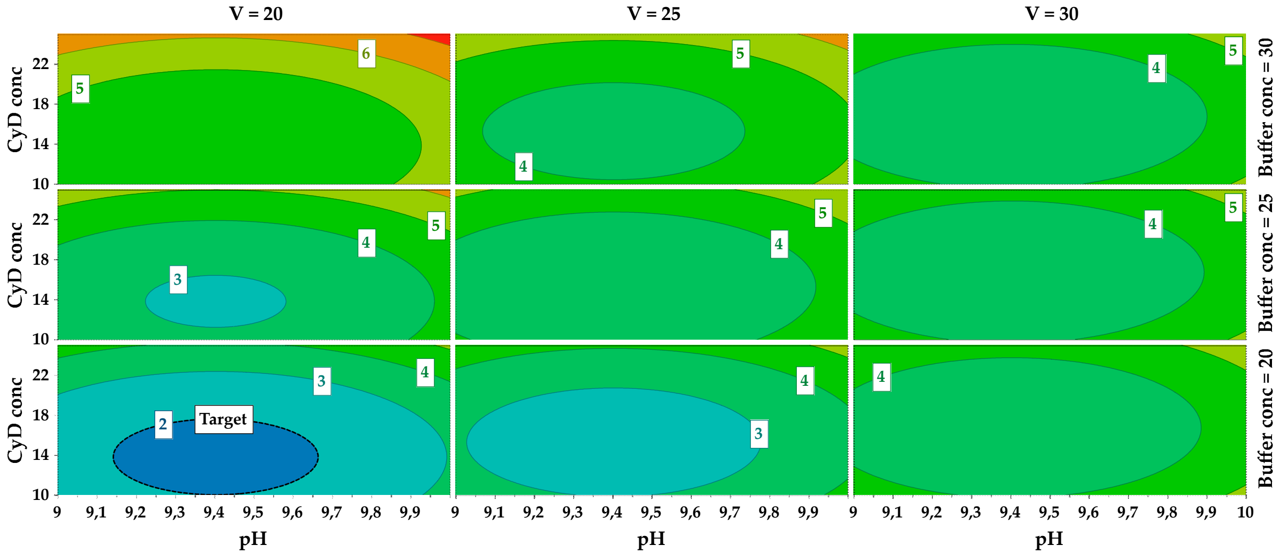

Figure S6. Resolution R5 contour plot.

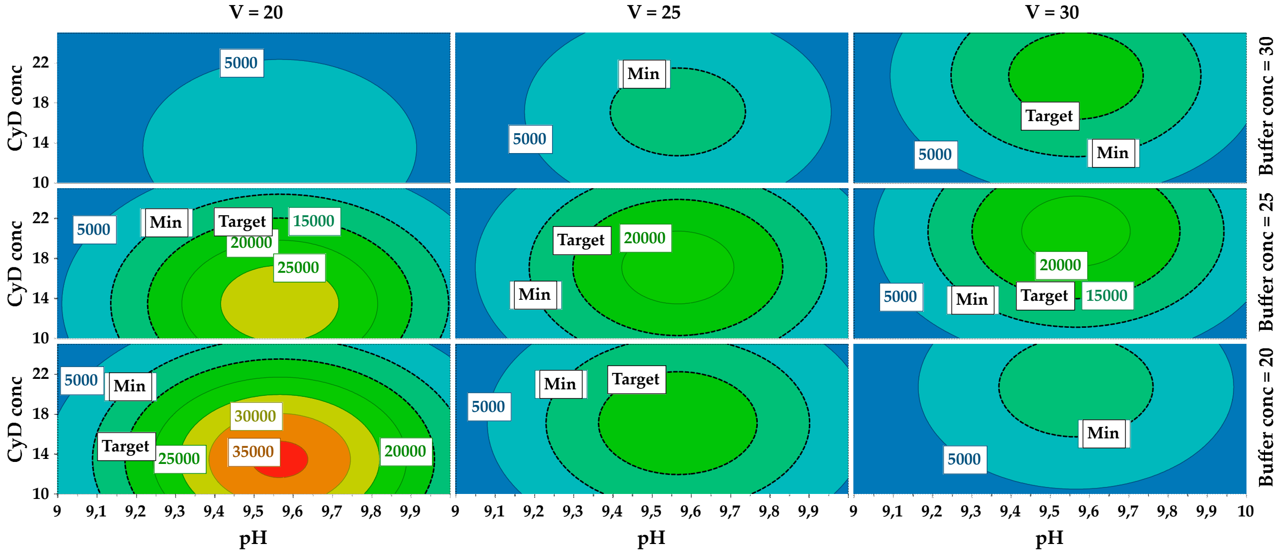

Figure S7. Efficiency N_{12} contour plot.

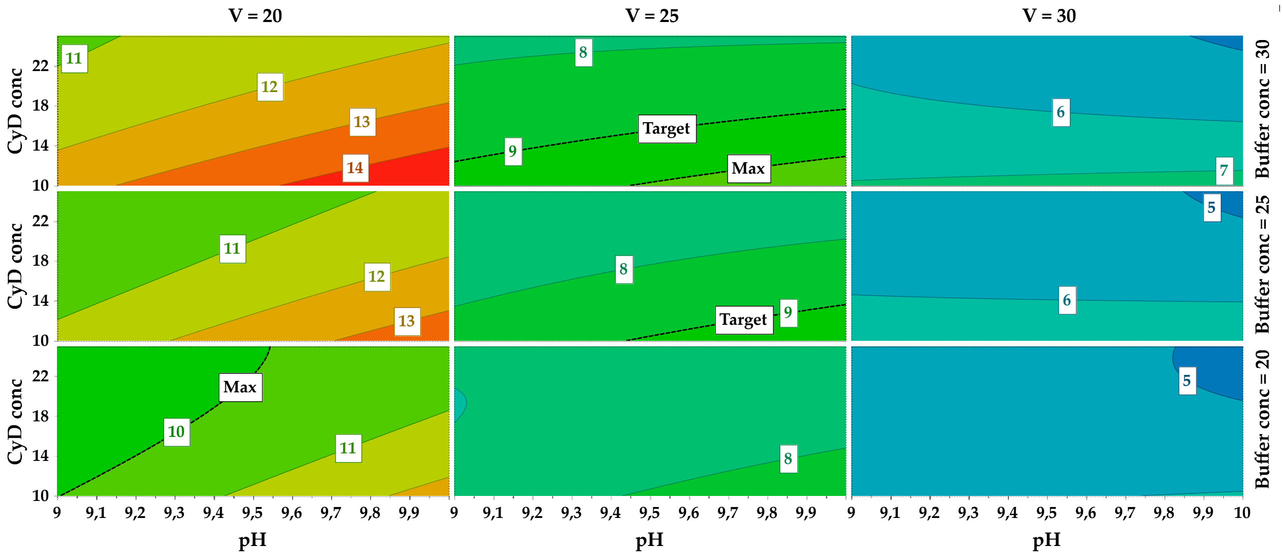

Figure S8. Analysis time contour plot.

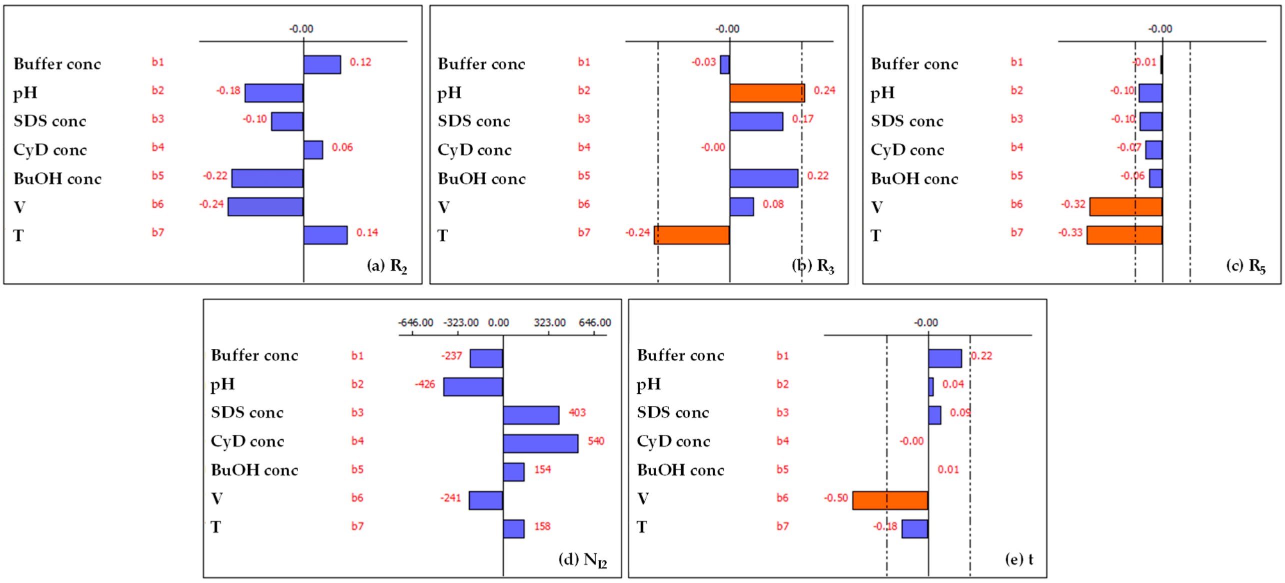

Figure S9. Robustness graphic analysis of effects. a) R_2, resolution I_1/I_2; (b), R_3, resolution I_2/I_3; (c) R_5, resolution TMC/I_4; (d) N_{I2}, I_2 efficiency; (e) t, analysis time.

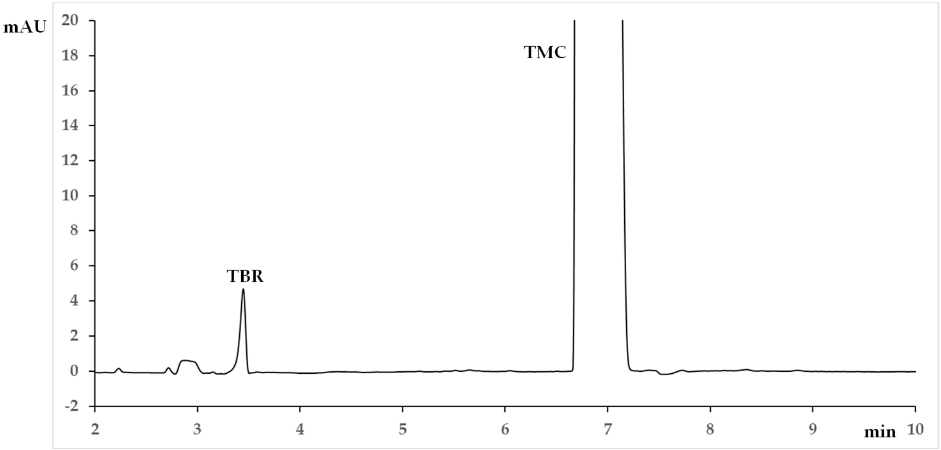

Figure S10. Electropherogram of the real sample Mesocain®.

Table S1. Quality parameters of the RSM models.

CMA	R^2	Q^2	Reproducibility
R_2	0.9152	0.7478	0.9642
R_3	0.9781	0.9172	0.9993
R_5	0.8257	0.4570	0.9965
N_{I2}	0.8778	0.4022	0.9055
t	0.9959	0.9823	0.9873

Table S2. Plackett-Burman Design for robustness testing.

No. Exp.	Buffer conc (mM)	pH	SDS conc (mM)	CyD conc (mM)	BuOH conc (% v/v)	V (kV)	T (°C)	R_2	R_3	R_5	N_{I2}	t (min)
1	25	9.80	67.0	19	1.10	24	21	2.15	3.08	4.23	13623	9.11
2	21	9.80	67.0	21	0.90	26	21	2.01	2.86	3.57	14386	7.64
3	21	9.60	67.0	21	1.10	24	23	2.68	2.17	3.64	16344	8.23
4	25	9.60	63.0	21	1.10	26	21	2.37	2.41	3.85	14267	7.85
5	21	9.80	63.0	19	1.10	26	23	1.92	2.47	3.14	13124	7.12
6	25	9.60	67.0	19	0.90	26	23	2.77	1.83	3.25	14002	7.66
7	25	9.80	63.0	21	0.90	24	23	3.20	1.81	3.73	13903	8.56
8	21	9.60	63.0	19	0.90	24	21	2.93	1.87	4.77	13834	8.40

R_2, resolution I_1/I_2; R_3, resolution I_2/I_3; R_5, resolution TMC/I_4; N_{I2}, I_2 efficiency; t, analysis time.

Table S3. Validation data.

	TMC	I_1	I_2	I_3	I_4
DL (mg mL^{-1})	n.a.	0.0035 (0.07%)	0.0037 (0.07%)	0.0008 (0.02%)	0.0010 (0.02%)
QL (mg mL^{-1})	n.a.	0.0050 (0.10%)	0.0050 (0.10%)	0.0030 (0.06%)	0.0030 (0.06%)
Working Range (mg mL^{-1})	3.0000-6.0000	0.0050-0.0500	0.0050-0.0500	0.0030-0.0500	0.0030-0.0500
Slope a	8.7633	16.9133	16.1627	31.3573	19.2059
Intercept b	25.1253	0.0209	0.0220	0.0360	0.0220
s_a	0.1222	0.3999	0.3481	0.5292	0.2358
s_b	0.5641	0.0126	0.0110	0.0164	0.0073
$s_{x/y}$	0.4338	0.0212	0.0184	0.0290	0.0129
R^2	0.9981	0.9944	0.9954	0.9972	0.9985
Accuracy and precision					
QL concentration level (mg mL^{-1})	n.a.	0.0050	0.0050	0.0030	0.0030
Recovery	n.a.	101.06±11.10%	104.09±14.90%	95.32±16.16%	105.39±14.69%
RSD	n.a.	4.42%	5.76%	6.82%	5.61%
Low concentration level (mg mL^{-1})	3.3000	0.0100	0.0100	0.0080	0.0080
Recovery	99.49±2.77%	95.14±8.77%	95.52±10.02%	101.78±8.43%	104.83±1088%
RSD	1.12%	3.71%	4.22%	3.33%	4.18%
Medium concentration level (mg mL^{-1})	4.5000	0.0280	0.0280	0.0260	0.0260
Recovery	101.64±3.18%	96.46±9.51%	97.04±12.50%	95.46±10.54%	95.98±7.92%
RSD	1.26%	3.97%	5.18%	4.44%	3.32%
High concentration level (mg mL^{-1})	5.7000	0.0450	0.0450	0.0450	0.0450
Recovery	100.75±4.03%	104.41±7.73%	96.79±6.20%	102.31±8.63%	102.27±4.00%
RSD	1.61%	2.98%	2.58%	3.40%	1.57%

[1] n.a., not assessed for the API. Regression equation for the working range: *y*, ratio of peak corrected area of the analyte to internal standard; s_a, standard deviation of the slope; s_b, standard deviation of the intercept; $s_{x/y}$, standard deviation of the residuals; R^2, coefficient of determination. For Accuracy, the recovery percentage was calculated from the formula $m \pm (t \cdot SD)/(n)^{0.5}$, where *m* is the average of the recovery values, *SD* is the standard deviation and *n* is the number of replicates (n=3). For Precision, the RSD values were calculated as $(SD/m) \cdot 100$.

Table S4. Within-day and between-day instrumental repeatability.

	Within-day RSD (n=6)		Total RSD (n=18)
**Compound**	**Day 1**	**Day 3**	**Days 1-2-3**
Corrected peak areas (analyte to internal standard)			
TMC	0.65%	0.82%	1.05%
I_1	1.94%	1.97%	1.65%
I_2	1.41%	0.95%	1.34%
I_3	1.39%	1.68%	2.03%
I_4	2.42%	2.41%	2.22%
Analysis time	1.45%	2.11%	2.03%

Table S5. Real sample analysis.

	CE method		UPLC method	
**Sample**	**Percentage of label claim**	**RSD**	**Percentage of label claim**	**RSD**
Sample 01: DS	100.43±3.37%	2.11%	100.40±0.16%	0.10%
Sample 02: DS	100.49±3.52%	2.20%	100.04±0.26%	0.17%
Sample 03: DP	98.46±2.87%	1.83%	100.02±0.95%	0.59%
Sample 04: DP	101.89±2.60%	1.61%	100.51±0.84%	0.53%

Table S6. Comparison between the CE method and the UPLC method.

Sample	CE method	UPLC method
Analysis time	8 min	20 min
QL for the impurities	0.060-0.100%	0.013-0.023%
DL for the impurities	0.020-0.070%	0.004-0.007%
Volume of organic solvent for one sample	About 20 µL of *n*-butanol	About 80 mL of methanol/acetonitrile
